# Multimodal Prediction of Progression Toward Brain Death After Out-of-Hospital Cardiac Arrest

**DOI:** 10.3390/jcm15145751

**Published:** 2026-07-22

**Authors:** Jae Hun Oh, Jisu Kim, Jong Ho Zhu, Mi Kyong Kwon, Seung Pill Choi, Hyo Joon Kim, Kiwook Kim, Hwan Song, Soo Hyun Kim

**Affiliations:** 1 Department of Emergency, Eunpyeong St. Mary’s Hospital, College of Medicine, The Catholic University of Korea, Seoul 03312, Republic of Korea; emojh@catholic.ac.kr (J.H.O.); 6fold@naver.com (J.K.); kingmonst2r@gmail.com (J.H.Z.); artient@hanmail.net (M.K.K.); emvic98@catholic.ac.kr (S.P.C.); 2 Department of Emergency Medicine, Seoul St. Mary’s Hospital, College of Medicine, The Catholic University of Korea, Seoul 06591, Republic of Korea; khjoon0110@gmail.com; 3 Department of Emergency Medicine, Uijeongbu St. Mary’s Hospital, College of Medicine, The Catholic University of Korea, Uijeongbu-si 11765, Republic of Korea; kiwookkim@catholic.ac.kr; 4 Department of Emergency Medicine, St. Vincent’s Hospital, College of Medicine, The Catholic University of Korea, Suwon-si 16247, Republic of Korea; cmcmdsong@gmail.com

**Keywords:** heart arrest, brain death, prognosis, targeted temperature management, neuron-specific enolase, gray-to-white matter ratio

## Abstract

**Background/Objectives**: Some patients with severe hypoxic–ischemic brain injury after out-of-hospital cardiac arrest (OHCA) progress toward brain death, a trajectory not adequately captured by the conventional classification of favorable versus unfavorable neurological outcomes. We developed and internally evaluated a multimodal model combining quantitative brain computed tomography (CT), serum neuron-specific enolase (NSE) at 48 h, and clinical variables to predict operationally defined progression toward brain death (PTBD). **Methods**: This multicenter retrospective secondary analysis used prospectively collected data from the Korean Hypothermia Network registry. Adult comatose OHCA survivors treated with targeted temperature management between October 2015 and December 2020 were included. Multivariable logistic regression models were developed in the total cohort and in patients with poor neurological outcomes. Model performance was assessed using discrimination, calibration, the Brier score, and bootstrap internal validation. **Results**: Of 468 patients assessed, 376 were included; their mean age was 58.7 years, and 269 (71.5%) were male. Seventy-four patients (19.7%) met the operational definition of PTBD. In the total cohort, younger age, non-shockable rhythm, low gray-to-white matter ratio (GWR ≤ 1.19), and higher NSE at 48 h were independently associated with PTBD. Among 288 patients with poor neurological outcomes, younger age, low GWR, and higher NSE at 48 h remained independent predictors. The total-cohort model had an AUC of 0.895 and an optimism-corrected AUC of 0.890. Its AUC was higher than that of NSE at 48 h (*p* < 0.001) but not significantly different from that of GWR alone (*p* = 0.054). In the poor-outcome subgroup, the model had an AUC of 0.864 and an optimism-corrected AUC of 0.858 and significantly outperformed both GWR (*p* = 0.012) and NSE at 48 h (*p* < 0.001). **Conclusions**: PTBD represents a clinically distinguishable trajectory among patients with poor neurological outcomes after OHCA. A multimodal model using information available within 48 h demonstrated good internally validated performance and may support early risk stratification before definitive neuroprognostication. External validation is required before clinical implementation.

## 1. Introduction

Out-of-hospital cardiac arrest (OHCA) remains a leading cause of mortality and severe neurological disability worldwide. A systematic review reported an overall survival-to-discharge rate of approximately 9% after OHCA, while a nationwide Korean study reported a 1-year survival rate of 8.2%, corresponding to mortality exceeding 90% [1,2]. Despite advances in post-resuscitation care, including targeted temperature management (TTM), hypoxic–ischemic brain injury (HIBI) continues to account for most in-hospital deaths among comatose survivors after return of spontaneous circulation (ROSC) [3,4]. Current international guidelines recommend multimodal neuroprognostication integrating neurological examination, electrophysiological testing, neuroimaging, and serum biomarkers to estimate neurological prognosis after cardiac arrest [3,5]. However, these approaches are primarily designed to distinguish patients with favorable from unfavorable neurological outcomes.

Importantly, poor neurological outcome is not a homogeneous endpoint. While some patients survive with severe neurological disability or die from systemic complications, others follow a clinical course characterized by progressive cerebral edema, loss of brainstem function, and eventual progression toward brain death (PTBD). Early identification of this subgroup is clinically relevant because it may allow clinicians to anticipate catastrophic neurological deterioration and intensify neurological surveillance before definitive multimodal neuroprognostication is performed. Nevertheless, current neuroprognostication strategies do not specifically distinguish patients with PTBD from those with non-PTBD poor neurological outcomes.

Several recent studies have demonstrated that early prediction of PTBD after OHCA is feasible. Madelaine et al. developed the Brain Death after Cardiac Arrest (BDCA) score using demographic, clinical, and laboratory variables obtained within 24 h after ROSC [6]. Kitlen et al. subsequently proposed the Brain Death Risk (BDR) score incorporating arrest characteristics, qualitative computed tomography (CT) findings, neurological examination, and age [7]. Lee et al. further demonstrated the prognostic value of early serum neuron-specific enolase (NSE) concentrations and absent pupillary light reflex in a Korean single-center cohort [8]. Collectively, these studies established that patients progressing toward BD can be identified during the early post-resuscitation period. However, the extent to which such risk stratification can contribute to clinical management before definitive neurological assessment remains to be established.

However, important gaps remain. Existing models rely predominantly on clinical variables or qualitative neuroimaging findings, with limited integration of quantitative imaging and biochemical markers reflecting complementary aspects of HIBI. In particular, few studies have evaluated whether NSE measured at a predefined post-ROSC time point provides complementary information when combined with quantitative neuroimaging and routinely available clinical variables. Furthermore, most derivation cohorts originated from healthcare systems where withdrawal of life-sustaining treatment (WLST) is routinely practiced. Because WLST frequently precedes the natural evolution toward BD, treatment withdrawal may influence the observed incidence, timing, and clinical characteristics of patients progressing toward BD.

South Korea provides a unique setting to investigate the natural evolution of severe HIBI after OHCA. Although the Act on Decisions on Life-Sustaining Treatment was implemented in 2018, withdrawal of life-sustaining treatment remains relatively uncommon because of legal, cultural, and family-related factors [9,10]. Consequently, patients with devastating neurological injury are more likely to continue receiving life-sustaining treatment during the acute post-resuscitation period, allowing the PTBD trajectory to be evaluated with less influence from early treatment withdrawal.

Quantitative gray-to-white matter ratio (GWR) measured on early brain computed tomography (CT) reflects the severity of diffuse cerebral edema, whereas serum NSE reflects neuronal injury evolving after ROSC [11,12]. Although NSE measured at 48 h is not a prehospital or immediate post-ROSC marker, it remains available before definitive multimodal neuroprognostication, which is generally deferred until at least 72 h after rewarming in patients treated with TTM and after major confounders have been excluded [3,5]. In this clinical context, information available within the first 48 h may support early identification of patients following a PTBD trajectory. We hypothesized that integrating these complementary structural and biochemical markers with established clinical variables would improve early identification of patients progressing toward BD compared with individual predictors alone.

Therefore, we aimed to develop and internally evaluate a multimodal prediction model integrating quantitative brain CT, serum NSE measured at 48 h, and clinical variables for identifying patients progressing toward BD in a multicenter cohort of comatose OHCA survivors treated with TTM. Although the model was not intended to provide prehospital or immediate post-ROSC prediction, all included predictors were available within the first 48 h, before definitive guideline-recommended neuroprognostication. Rather than considering poor neurological outcome as a single endpoint, we specifically distinguished patients progressing toward BD from those with poor neurological outcomes without BD. Analyses were performed in both the total cohort and the clinically relevant subgroup of patients with poor neurological outcomes. The subgroup analysis specifically examined whether PTBD could be distinguished from other unfavorable neurological trajectories.

## 2. Materials and Methods

### 2.1. Study Design

This study was a multicenter retrospective secondary analysis of prospectively collected data from the Korean Hypothermia Network (KORHN) registry. Four university-affiliated tertiary care hospitals participating in the KORHN registry were included. The registry prospectively and consecutively enrolled post-cardiac arrest patients treated with TTM using standardized case report forms and predefined data definitions. The design and data collection procedures of the KORHN registry have been described previously [13]. For the present study, we retrospectively analyzed adult patients with OHCA who underwent TTM between October 2015 and December 2020. The reporting of this study was guided by the Transparent Reporting of a multivariable prediction model for Individual Prognosis Or Diagnosis (TRIPOD) statement.

### 2.2. Study Population

Eligible patients were adults (≥18 years) with non-traumatic OHCA who achieved ROSC and subsequently received TTM according to institutional protocols. Patients were excluded if they did not undergo TTM, experienced only in-hospital cardiac arrest, had unavailable brain CT or quantitative GWR data, had incomplete NSE measurements required for the present analysis, or had missing data for other prespecified variables.

**Figure 1:**
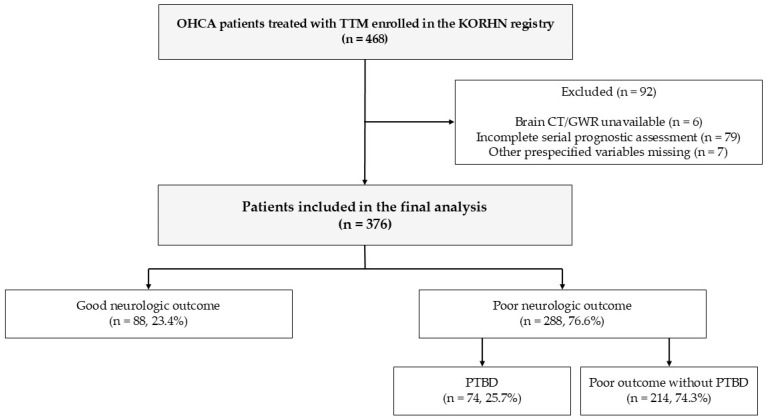
Flow diagram of patient inclusion. During the study period, 468 patients with OHCA treated with TTM were assessed. After exclusion of patients with unavailable brain CT or quantitative GWR data (*n* = 6), incomplete NSE measurements (*n* = 79), or missing data for other prespecified variables (*n* = 7), 376 patients were included in the final analysis. OHCA, out-of-hospital cardiac arrest; TTM, targeted temperature management; CT, computed tomography; GWR, gray-to-white matter ratio; NSE, neuron-specific enolase.

During the study period, 468 patients with OHCA who completed TTM were identified in the participating-center registry cohort. Of these, 6 were excluded because brain CT or quantitative GWR data were unavailable, 79 because of incomplete NSE measurements, and 7 because of missing data for other prespecified variables. The final analytic cohort therefore included 376 patients (Figure 1).

The presumed cause of cardiac arrest was classified according to the standardized registry definitions. A cardiac etiology was assigned when a primary cardiac cause was considered most likely and no definite non-cardiac cause was identified. Arrests attributable to an identifiable non-cardiac cause, including asphyxial causes such as hanging, were categorized as non-cardiac. Traumatic cardiac arrests were not included.

### 2.3. Post-Cardiac Arrest Care and Targeted Temperature Management

Post-cardiac arrest care followed contemporary international guidelines and KORHN consensus protocols. TTM was initiated as early as possible after intensive care unit admission using either surface or intravascular cooling devices. Target temperature (typically 33–36 °C) was selected according to institutional practice and maintained for at least 24 h, followed by controlled rewarming to normothermia. Patients managed at a target temperature of 36 °C also underwent controlled transition to normothermia rather than further active warming beyond the normal temperature range. Hemodynamic support, mechanical ventilation, sedation, analgesia, and neuromuscular blockade were administered according to local protocols and the treating physician’s clinical judgment.

### 2.4. Operational Definition of Progression Toward Brain Death

Because formal legal determination of brain death was not systematically performed or available for all patients enrolled in the registry, the primary outcome of this study was operationally defined PTBD based on predefined neurological and electrophysiological criteria recorded in the registry.

Patients were classified as having PTBD when all of the following criteria were fulfilled after adequate discontinuation and washout of sedative, analgesic, and neuromuscular blocking agents:

(1) Electroencephalography (EEG) demonstrated findings compatible with severe cerebral inactivity, including electrocerebral inactivity or a markedly suppressed background pattern, as interpreted by an experienced neurologist or clinical neurophysiologist;

(2) No spontaneous respiratory effort was observed during mechanical ventilation;

(3) Repeated neurological examinations demonstrated complete absence of brainstem reflexes, including pupillary light reflex, corneal reflex, oculocephalic or oculovestibular reflexes, cough reflex, and gag reflex.

Adequate drug washout was determined by the treating team according to institutional protocols, considering the time elapsed since the last administration, expected drug half-life, cumulative exposure, body temperature, and hepatic or renal dysfunction that could delay drug clearance. Neurological assessment was performed after residual sedative and neuromuscular blocking effects were considered unlikely to confound the examination.

Neurological examinations and EEG interpretation were performed according to institutional post-cardiac arrest protocols using the standardized definitions included in the KORHN registry. Although the registry provided common data definitions, the examinations and EEG interpretations were performed locally and were not centrally adjudicated.

This operational endpoint was predefined before the present analysis using standardized registry variables and applied uniformly across participating centers. It was designed to identify patients following a clinical course compatible with PTBD within the constraints of registry-based data collection and should not be interpreted as equivalent to formal legal determination of brain death. Ancillary cerebral perfusion studies, including positron emission tomography or perfusion imaging, were not required for this operational classification and were not systematically available in the registry. Patients who subsequently became organ donors underwent the separate national legal brain death determination process.

### 2.5. Data Collection and Variables

Clinical data were extracted from the KORHN registry and supplemented by review of electronic medical records at each participating center. Data were collected by trained investigators at each institution using standardized case report forms and predefined registry definitions. Registry data underwent routine checks for completeness and internal consistency, and discrepancies were reviewed against the source medical records at the participating center. Collected variables included demographic characteristics (age and sex), premorbid comorbidities (coronary artery disease, hypertension, diabetes mellitus, cerebrovascular disease, and chronic pulmonary disease), arrest-related variables (witnessed status, bystander cardiopulmonary resuscitation, initial rhythm, presumed etiology, and time from collapse to ROSC), immediate post-ROSC physiological and laboratory findings, and TTM-related variables (pre-induction shock, rearrest, arrhythmias, glycemic and electrolyte disturbances, and post-rewarming hyperthermia).

Variables considered for the regression analyses were selected from clinically relevant factors available in the registry and included age; witnessed arrest; bystander cardiopulmonary resuscitation; shockable initial rhythm; time to ROSC; cardiac etiology; hanging; initial pH, lactate, and glucose concentrations; pre-induction shock; Glasgow Coma Scale motor score > 1; presence of pupillary light reflex; convulsive movement; NSE concentrations at 24, 48, and 72 h; and low GWR. Comorbidities and other TTM-related variables were summarized descriptively and evaluated as potential confounders when clinically relevant.

Neuroprognostic variables included EEG findings, serum NSE concentrations measured at 24, 48, and 72 h after ROSC, and early brain CT findings. Quantitative GWR was measured on brain CT obtained within 2 h after ROSC using predefined regions of interest in the basal ganglia and cortical areas by two trained investigators blinded to the clinical data. The investigators assessed the CT images independently using a standardized region-of-interest method described previously. Attenuation was measured bilaterally in predefined gray- and white-matter regions at the basal ganglia, centrum semiovale, and high-convexity levels using a 10 mm^2^ circular cursor. The basal ganglia GWR was calculated as (CN + PU)/(CC + PIC), and the cerebral GWR as (MC1 + MC2)/(MW1 + MW2). The average GWR was calculated as the mean of the basal ganglia and cerebral GWRs. For each patient, the mean of the average GWR values independently obtained by the two investigators was used in the analysis [14,15,16]. Serum NSE concentrations were measured using commercially available immunoassays at each participating institution, and values below the lower limit of detection were recorded as the corresponding detection limit. NSE values at 24, 48, and 72 h were examined in the univariable analyses; NSE at 48 h was retained in the final multivariable models. Thus, the final models incorporated a single predefined NSE time point rather than serial changes or NSE kinetics.

### 2.6. Outcome Measures

The primary outcome was operationally defined PTBD during the index hospitalization.

Secondary outcomes included:

(1) Good neurological outcome at 6 months after ROSC (CPC 1–2);

(2) Poor neurological outcome at 6 months (CPC 3–5);

(3) The proportion of patients in the PTBD group who ultimately underwent deceased organ donation.

For the subgroup analysis, patients with a poor neurological outcome were classified as having either PTBD or a non-PTBD poor neurological outcome.

Organ donation was defined as successful procurement of at least one solid organ following completion of the national legal brain death determination process.

### 2.7. Statistical Analysis

Continuous variables are presented as mean ± standard deviation or median with interquartile range, as appropriate, and categorical variables as frequencies and percentages. Comparisons across the three outcome groups were performed using one-way analysis of variance or the Kruskal–Wallis test for continuous variables and the chi-square test or Fisher’s exact test for categorical variables, as appropriate. Comparisons between patients with PTBD and those with non-PTBD poor neurological outcomes were performed using Student’s *t*-test or the Mann–Whitney U test for continuous variables and the chi-square test or Fisher’s exact test for categorical variables.

Univariable logistic regression analysis was first performed to examine the association of each candidate predictor with PTBD. Multivariable logistic regression models were then constructed for the total cohort and the subgroup of patients with poor neurological outcomes. Candidate predictors were selected based on clinical relevance, the previous literature, univariable associations, the number of outcome events, and potential collinearity. NSE measurements obtained at 24, 48, and 72 h were evaluated separately in the univariable analyses; NSE at 48 h was used in the final models to represent the biomarker component without simultaneously including correlated NSE measurements from multiple time points. Results are presented as odds ratios (ORs) and adjusted odds ratios (aORs) with 95% confidence intervals (CIs). 

Model discrimination was evaluated using the area under the receiver operating characteristic curve (AUC) with a 95% CI. The discriminative performance of quantitative GWR, NSE measured at 24, 48, and 72 h, and the final multimodal models was examined separately. Cut-off values maximizing the Youden index and cut-off values providing 100% specificity were reported with their corresponding sensitivity and specificity. Pairwise comparisons of correlated AUCs between the final model and each individual predictor were performed using DeLong’s test. Internal validation was performed using bootstrap resampling with 1000 iterations. Model performance was summarized using the apparent and optimism-corrected AUC, calibration slope, calibration intercept, and Brier score. Forest plots and ROC curves were generated using the ggplot2 package (version 4.0.3).

Because the cohort size was fixed by the number of eligible patients available in the registry during the study period, no separate a priori sample-size calculation was performed. All consecutively registered patients who met the eligibility criteria and had the data required for the present analysis were included. Group comparisons of baseline and clinical variables and pairwise AUC comparisons were considered descriptive and exploratory; therefore, no formal adjustment for multiple comparisons was applied. Statistical inference regarding the primary outcome was based principally on the regression models and their 95% CIs rather than on isolated unadjusted group-comparison *p* values. All statistical analyses were performed using R software (version 4.5.2; R Foundation for Statistical Computing, Vienna, Austria). A two-sided *p* value < 0.05 was considered statistically significant.

### 2.8. Ethical Considerations

This study was conducted in accordance with the principles of the Declaration of Helsinki. The protocol for the present retrospective secondary analysis was approved by the Institutional Review Board of Eunpyeong St. Mary’s Hospital on 30 December 2021 (IRB No. PC21RISI0229). Because of the retrospective nature of the study and the use of de-identified registry data, the requirement for written informed consent from patients or their legal surrogates was waived by the Institutional Review Board.

## 3. Results

### 3.1. Study Population

During the study period, 468 patients with OHCA treated with TTM were assessed for eligibility. Of these, 6 were excluded because brain CT or quantitative GWR data were unavailable, 79 because of incomplete NSE measurements, and 7 because of missing data for other prespecified variables. The final analytic cohort comprised 376 patients (Figure 1). At 6 months after ROSC, 88 patients (23.4%) had a good neurological outcome and 288 (76.6%) had a poor neurological outcome. Among the 288 patients with poor neurological outcomes, 214 (56.9% of the total cohort) were classified as having a non-PTBD poor neurological outcome, and 74 (19.7%) met the operational definition of PTBD. Among the 74 patients with PTBD, 31 (41.9%) subsequently underwent deceased organ donation following completion of the national legal brain death determination process.

### 3.2. Baseline and Clinical Characteristics

Baseline demographic and resuscitation characteristics according to neurological outcome groups are summarized in Table 1.

Patients with non-PTBD poor neurological outcomes were older than both patients with good neurological outcomes and those with PTBD. Within the poor neurological outcome subgroup, patients with PTBD were younger than those without PTBD (51.7 ± 16.8 vs. 64.0 ± 15.2 years, *p* < 0.001).

Resuscitation characteristics differed across the three groups. Compared with patients with non-PTBD poor neurological outcomes, those with PTBD less frequently had a shockable initial rhythm (9.5% vs. 21.5%, *p* = 0.033), had a longer time to ROSC (42.3 ± 19.0 vs. 35.3 ± 21.5 min, *p* = 0.003), and less frequently had a presumed cardiac etiology (32.4% vs. 57.5%, *p* < 0.001). Hanging was more frequent among patients with PTBD than among those with non-PTBD poor neurological outcomes (36.5% vs. 8.9%, *p* < 0.001). Immediately after ROSC, a Glasgow Coma Scale motor score >1 was less frequent in the PTBD group than in the non-PTBD poor neurological outcome group (4.1% vs. 13.6%, *p* = 0.043). Patients with PTBD also had a lower initial pH (*p* = 0.013) and a higher initial lactate concentration (11.9 ± 5.4 vs. 10.4 ± 5.8 mmol/L, *p* = 0.025). Mean arterial pressure, initial glucose concentration, and the presence of pupillary light reflex did not differ significantly between the two poor-outcome groups.

Several TTM-related variables differed across the three outcome groups, including pre-induction shock, induction time, post-rewarming hyperthermia, rearrest, hypoglycemia, and hyperglycemia (Table 1). However, none of the evaluated TTM-related variables differed significantly between patients with PTBD and those with non-PTBD poor neurological outcomes.

### 3.3. Univariable and Multivariable Analyses for PTBD in the Total Cohort

In the total cohort, younger age, unwitnessed arrest, non-shockable rhythm, longer time to ROSC, non-cardiac etiology, hanging, lower initial pH, higher initial lactate and glucose concentrations, pre-induction shock, a Glasgow Coma Scale motor score of 1, absence of pupillary light reflex, higher NSE concentrations at 24, 48, and 72 h, and low GWR were associated with PTBD in the univariable analyses (Table 2).

**Figure 2:**
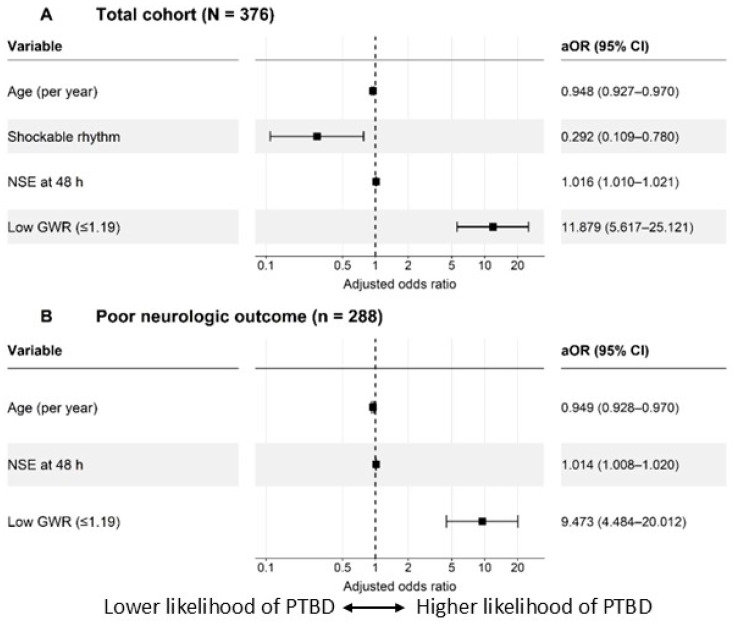
Forest plots of multivariable logistic regression analyses for progression toward brain death. Panel (**A**) shows the final model in the total cohort (*n* = 376), and Panel (**B**) shows the final model restricted to patients with poor neurological outcomes (*n* = 288). Squares indicate adjusted odds ratios, and horizontal lines represent 95% confidence intervals. The vertical dashed line indicates an odds ratio of 1.0. Values to the right indicate a greater likelihood of PTBD, whereas values to the left indicate a lower likelihood of PTBD. GWR, gray-to-white matter ratio; NSE, neuron-specific enolase; PTBD, progression toward brain death.

In the final multivariable model, younger age (aOR per year, 0.948; 95% CI, 0.927–0.970; *p* < 0.001), non-shockable initial rhythm (aOR for shockable rhythm, 0.292; 95% CI, 0.109–0.780; *p* = 0.014), higher NSE at 48 h (aOR per unit, 1.016; 95% CI, 1.010–1.021; *p* < 0.001), and low GWR (≤1.19; aOR, 11.879; 95% CI, 5.617–25.121; *p* < 0.001) remained independently associated with PTBD (Table 2 and Figure 2A).

The final model for the total cohort was expressed as follows:

$$\begin{array}{ccc} logit(P_{PTBD}) & = & -0.723+2.475(low\;GWR)-1.232(shockable\;rhythm)-0.053(age)\\ & & +0.015(NSE\;at\;48\;h) \end{array}$$

where low GWR and shockable rhythm were coded as binary variables and $P_{PTBD}=1/[1+exp\{-logit(P_{PTBD})\}]$.

The model had an apparent AUC of 0.895 and an optimism-corrected AUC of 0.890 after bootstrap validation. The calibration slope was 0.956, the calibration intercept was −0.030, and the Brier score was 0.093.

### 3.4. Univariable and Multivariable Analyses in Patients with Poor Neurological Outcomes

Among the 288 patients with poor neurological outcomes, younger age, non-shockable rhythm, longer time to ROSC, non-cardiac etiology, hanging, lower initial pH, higher initial lactate concentration, a Glasgow Coma Scale motor score of 1, higher NSE concentrations at 24, 48, and 72 h, and low GWR were associated with PTBD in the univariable analyses (Table 3).

In the final multivariable model, younger age (aOR per year, 0.949; 95% CI, 0.928–0.970; *p* < 0.001), higher NSE at 48 h (aOR per unit, 1.014; 95% CI, 1.008–1.020; *p* < 0.001), and low GWR (≤1.19; aOR, 9.473; 95% CI, 4.484–20.012; *p* < 0.001) remained independently associated with PTBD (Table 3 and Figure 2B). Shockable rhythm was associated with PTBD in the univariable analysis but was not retained in the final multivariable model.

The final model for patients with poor neurological outcomes was expressed as follows: logit($P_{PTBD}$) = −0.530 + 2.248 (low GWR) − 0.053 (age) + 0.014 (NSE at 48 h), where low GWR was coded as 1 for GWR ≤1.19 and 0 for GWR >1.19, and $P_{PTBD}=1/[1+exp\{-logit(P_{PTBD})\}]$.

The model demonstrated an apparent AUC of 0.864 and an optimism-corrected AUC of 0.858. The calibration slope was 0.960, the calibration intercept was −0.022, and the Brier score was 0.122.

### 3.5. Discriminative Performance of GWR, NSE, and the Multimodal Models

**Figure 3:**
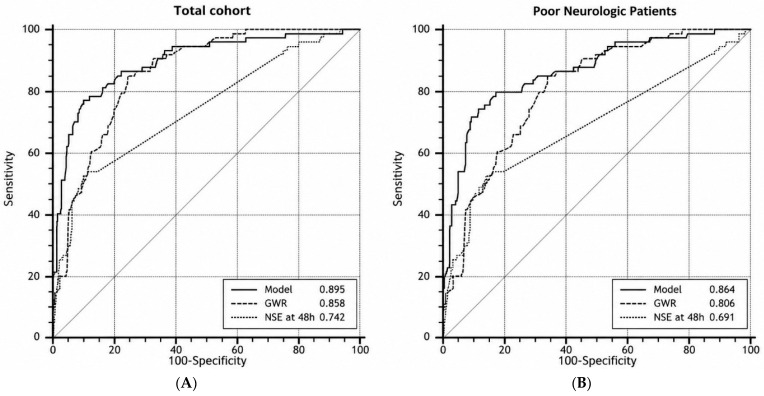
Receiver operating characteristic curves for prediction of progression toward brain death. ROC curves compare the discriminative performance of the final multimodal model with quantitative brain CT GWR and serum NSE measured at 48 h. Panel (**A**) shows the total cohort (*n* = 376), and Panel (**B**) shows the subgroup of patients with poor neurological outcomes (*n* = 288). GWR, gray-to-white matter ratio; NSE, neuron-specific enolase; ROC, receiver operating characteristic; PTBD, progression toward brain death.

The discriminative performance of quantitative GWR, NSE measured at 24, 48, and 72 h, and the final multimodal models is presented in Table 4 and Figure 3.

In the total cohort, the final multimodal model had an AUC of 0.895 (95% CI, 0.860–0.924), compared with 0.858 (95% CI, 0.819–0.892) for quantitative GWR and 0.727, 0.742, and 0.708 for NSE at 24, 48, and 72 h, respectively. Pairwise comparisons using DeLong’s test showed that the final model had significantly greater discrimination than NSE at all three time points (all *p* < 0.001), whereas the difference between the final model and GWR alone did not reach statistical significance (*p* = 0.054). The final model demonstrated a sensitivity of 77.3% and a specificity of 90.1% at the selected cut-off.

Among patients with poor neurological outcomes, the final model had an AUC of 0.864 (95% CI, 0.819–0.901), compared with 0.806 (95% CI, 0.756–0.850) for quantitative GWR and 0.684, 0.691, and 0.661 for NSE at 24, 48, and 72 h, respectively. In this subgroup, the final model demonstrated significantly greater discrimination than GWR alone (*p* = 0.012) and NSE at all three time points (all *p* < 0.001). The final model demonstrated a sensitivity of 74.3% and a specificity of 88.3% at the selected cut-off. Detailed cut-off values and thresholds yielding 100% specificity are presented in Table 4.

## 4. Discussion

### 4.1. Main Findings

In this multicenter study of comatose survivors of OHCA treated with TTM, we developed and evaluated a multimodal prediction model for operationally defined PTBD. The principal finding of this study is that, among patients with poor neurological outcomes, progression toward brain death could be distinguished from other unfavorable neurological trajectories using early clinical, radiologic, and biomarker findings. These results suggest that PTBD may represent a distinct neurological trajectory rather than merely the extreme end of overall neurological injury severity. Patients in the PTBD group demonstrated a characteristic pattern of profound structural brain injury, reflected by markedly reduced GWR, together with neuronal injury reflected by elevated 48 h NSE concentrations. In the total cohort, the final model significantly outperformed NSE at all three time points, although its AUC did not differ significantly from that of GWR alone. In contrast, among patients with poor neurological outcomes, the final model demonstrated significantly greater discrimination than both GWR and NSE at all three time points. The models also showed limited optimism and satisfactory calibration on bootstrap internal validation.

Current neuroprognostication strategies are primarily designed to distinguish favorable from unfavorable neurological outcomes after cardiac arrest and appropriately guide prognostic assessment and clinical decision-making [1,5,17,18]. However, patients with poor neurological outcomes represent a biologically heterogeneous population. Some survive with severe neurological disability, others die from systemic complications, whereas a subset follow a clinical course characterized by progressive cerebral edema, loss of brainstem function, and eventual PTBD. Our findings suggest that these groups should not be regarded as clinically equivalent because they differ in both the severity and evolution of hypoxic–ischemic brain injury. Early identification of patients following this trajectory therefore represents a complementary objective of multimodal neuroprognostication rather than merely another approach to predicting poor neurological outcome.

Approximately one-fifth of patients in our cohort met the operational definition of PTBD, a proportion comparable to previous multicenter reports despite differences in healthcare systems and patient characteristics [6,7,19]. Among these patients, fewer than half ultimately underwent deceased organ donation. Although organ donation was not the primary focus of this study, this observation underscores the clinical importance of understanding the natural evolution of catastrophic hypoxic–ischemic brain injury beyond conventional neurological outcome assessment [15,20].

### 4.2. Comparison with Previous Studies

Our findings should be interpreted in the context of several recent studies that have substantially advanced early prediction of brain death after OHCA. Rather than challenging these previous models, the present study extends existing evidence by addressing several important methodological and clinical limitations.

Madelaine and colleagues first demonstrated that early prediction of PTBD was feasible through development of the BDCA score using demographic characteristics together with clinical and laboratory variables available within the first 24 h after ROSC [6]. Although the BDCA score showed excellent discrimination, it did not incorporate objective neuroimaging.

Kitlen and colleagues subsequently developed the BDR score by integrating arrest characteristics, neurological examination, qualitative CT findings, and age [7]. Their work represented an important advance by incorporating neuroimaging into early prediction. However, CT abnormalities were assessed qualitatively according to radiology reports rather than quantitatively. By contrast, quantitative GWR provides a continuous and reproducible measure of diffuse cerebral edema that more directly reflects the severity of early hypoxic–ischemic injury.

These findings are also consistent with a previous Korean study of OHCA patients treated with TTM, which reported the feasibility of predicting brain death after cardiac arrest but did not focus on an integrated multimodal prediction strategy [21]. Lee et al. demonstrated that early serum NSE concentrations and absent pupillary light reflex independently predicted PTBD in a Korean single-center cohort [8]. While consistent with our findings regarding the importance of biochemical and neurological markers, that study did not incorporate quantitative CT measurements or evaluate integration of multiple prognostic modalities.

The timing of the present model should be interpreted in the context of post-cardiac arrest neuroprognostication. Although NSE at 48 h is not a prehospital or immediate post-ROSC marker, it is available before definitive multimodal neuroprognostication, which is generally deferred until at least 72 h after ROSC or rewarming and after major confounders have been excluded [3,5]. Thus, the present model supports early risk stratification during the acute post-resuscitation period rather than immediate prediction after ROSC.

The present study extends these previous investigations in three important respects. First, we integrated structural neuroimaging, NSE measured at 48 h, and established clinical variables into a unified multimodal prediction model, consistent with current international recommendations emphasizing multimodal neuroprognostication [3,5,17,18]. Second, our study was performed within a multicenter Korean registry in a healthcare environment where WLST remains relatively uncommon. Because early treatment withdrawal frequently interrupts the natural progression toward brain death in many healthcare systems, our cohort provides an opportunity to characterize this clinical trajectory with less confounding from WLST [9,10]. Third, rather than considering poor neurological outcome as a single endpoint, we specifically distinguished patients meeting the operational definition of PTBD from other patients with poor neurological outcomes. We believe this distinction is clinically meaningful because these patients differ not only in prognosis but also in the biological evolution of brain injury and subsequent clinical management. However, because the variables required to calculate the previous scores were not completely available in our registry, the present study did not directly compare its model with the BDCA or BDR scores. Therefore, our findings should not be interpreted as demonstrating superiority over these existing models.

### 4.3. Biological Interpretation

The clinical characteristics associated with PTBD in the univariable analyses are biologically plausible and reflect the severity of hypoxic–ischemic brain injury. Non-cardiac etiologies, particularly asphyxial cardiac arrest, may produce prolonged global cerebral hypoxia before circulatory collapse, whereas prolonged time to ROSC reflects cumulative ischemic burden [4]. However, non-cardiac etiology, hanging, and time to ROSC were not retained in the final multivariable models. Their univariable associations may therefore be mediated, at least in part, by the severity of cerebral injury more directly reflected by GWR and NSE.

Younger age was independently associated with PTBD in both the total cohort and the poor neurological outcome subgroup. Although older age is generally associated with poor neurological recovery after cardiac arrest, older patients with unfavorable outcomes may be more likely to die from systemic complications or remain severely disabled without progressing toward brain death. In contrast, younger patients with profound cerebral injury may retain extracerebral organ function long enough to manifest the PTBD trajectory. This interpretation remains hypothesis-generating and requires confirmation in external cohorts.

Shockable rhythm was independently associated with a lower likelihood of PTBD in the total cohort but was not retained in the poor neurological outcome subgroup model. Its contribution in the total cohort may primarily reflect the association of a shockable rhythm with cardiac etiology and favorable neurological recovery. Once the analysis was restricted to patients with poor neurological outcomes, shockable rhythm provided limited additional information beyond age, GWR, and NSE.

Among imaging variables, quantitative GWR measured on early brain CT independently predicted PTBD. In the present study, CT was obtained within 2 h after ROSC. Loss of gray-white differentiation reflects diffuse cytotoxic edema resulting from failure of cellular energy metabolism and disruption of ionic homeostasis during severe hypoxic–ischemic brain injury [11,12]. Because these structural changes occur early after reperfusion, GWR provides an objective assessment of the severity of initial cerebral injury before many neurological signs become clinically apparent. Low GWR was the strongest independent predictor in both multivariable models.

Alternative CT-based approaches include qualitative assessment of loss of gray–white matter differentiation and sulcal effacement, regional GWR measurements, and automated segmentation or density-based quantification of cerebral edema. Qualitative interpretation is readily available but may be observer-dependent, whereas automated approaches may improve reproducibility but require further validation across different scanners and acquisition protocols. The average GWR used in this study integrates measurements from the basal ganglia and cerebral cortex but was not directly compared with these alternative approaches.

In contrast, serum NSE reflects ongoing neuronal destruction. Whereas early CT primarily captures structural injury immediately after reperfusion, elevated 48 h NSE concentrations likely reflect continued neuronal necrosis during the secondary phase of brain injury. NSE at 48 h remained independently associated with PTBD in both models, although NSE alone showed only modest discrimination. The aOR close to 1 reflects the effect associated with a 1-unit increase in NSE; based on the fitted coefficients, a 10-unit increase corresponds to an approximately 15–17% increase in the odds of PTBD.

The contribution of multimodal integration differed between the two analyses. In the total cohort, the final model did not significantly outperform GWR alone, suggesting that early quantitative GWR accounted for much of the discrimination across the full neurological outcome spectrum. However, among patients with poor neurological outcomes, the final model significantly outperformed both GWR and NSE alone. This observation further supports the principle of multimodal neuroprognostication advocated by current international guidelines, in which complementary structural, biochemical, and clinical information may provide more robust prognostic assessment than any individual modality alone [3,5,17,18].

Although NSE was measured at 24, 48, and 72 h, the final model incorporated only the 48 h value and did not evaluate serial changes or NSE kinetics. The present findings should therefore be interpreted as supporting the prognostic contribution of NSE measured at 48 h rather than the predictive value of temporal NSE dynamics.

### 4.4. Clinical Implications

The primary clinical implication of this study is improved early neurological risk stratification following OHCA. Current neuroprognostication appropriately focuses on identifying patients unlikely to achieve meaningful neurological recovery. Our findings suggest that patients progressing toward brain death represent a distinct subgroup within the broader population of poor neurological outcomes. Earlier recognition of this trajectory may facilitate anticipation of catastrophic neurological deterioration, optimize neurological monitoring, and support individualized intensive care planning during the early post-resuscitation period [3,5,17,18].

Importantly, our multimodal prediction model is not intended to replace guideline-recommended multimodal neuroprognostication or formal legal brain death determination. Rather, it should be considered a complementary tool for identifying patients who warrant closer neurological surveillance while definitive prognostic assessment continues according to established recommendations [3,5]. A high predicted probability of PTBD should not be interpreted as a diagnosis of brain death or used to support irreversible treatment decisions.

In healthcare systems where formal brain death determination is required before deceased organ donation, earlier recognition of patients following a PTBD trajectory may also facilitate multidisciplinary planning, including optimization of physiological support [20,22,23,24]. However, donor eligibility, referral to an organ procurement organization, and organ donation procedures should remain separate from the prediction model and proceed only after appropriate neurological evaluation and in accordance with applicable legal and institutional requirements. Decisions regarding continuation or withdrawal of life-sustaining treatment should remain independent of this prediction model and continue to follow established ethical and legal frameworks [9,10].

### 4.5. Strengths

This study has several notable strengths. First, it represents one of the largest multicenter Asian cohorts specifically evaluating PTBD after OHCA using standardized TTM protocols within the Korean Hypothermia Network registry. Second, unlike previous prediction models that relied predominantly on clinical variables or qualitative neuroimaging, we integrated quantitative GWR measurements with NSE measured at 48 h, combining complementary structural and biochemical markers of hypoxic–ischemic brain injury. Third, the relatively low frequency of WLST in Korea provided a unique opportunity to evaluate the natural progression toward brain death with less confounding from early treatment withdrawal than is encountered in many Western healthcare systems [9,10,19]. In addition, quantitative GWR can be readily measured from routinely acquired CT scans without additional cost or imaging procedures, supporting the practical applicability of the proposed model. The use of bootstrap internal validation and assessment of calibration and the Brier score, in addition to discrimination, further strengthened the evaluation of model performance.

### 4.6. Limitations

Several limitations should be acknowledged. First, although registry data were prospectively collected, the present analysis was retrospective and therefore remains susceptible to residual confounding and selection bias. In addition, 92 of the 468 patients assessed were excluded because of unavailable CT/GWR data, incomplete NSE measurements, or other missing variables, which may have introduced additional selection bias. Second, the primary outcome was operationally defined PTBD rather than formal legal determination of brain death in every patient. Consequently, some degree of outcome misclassification cannot be excluded, particularly among patients who deteriorated before completion of the formal determination process. Although standardized registry definitions were applied, neurological examinations and EEG interpretations were performed locally and were not centrally adjudicated. Third, although the relatively low frequency of WLST is an important strength for studying the natural history of severe hypoxic–ischemic brain injury, it may limit generalizability to healthcare systems where treatment withdrawal substantially alters the post-cardiac arrest clinical course. Fourth, quantitative GWR measurements were obtained by two trained investigators who were blinded to the clinical data using standardized methods and may not be immediately reproducible across all institutions. Although the investigators assessed the images independently and their measurements were averaged, manual GWR measurement may be affected by training, CT acquisition parameters, and scanner characteristics. Fifth, although the models demonstrated satisfactory performance after bootstrap internal validation, they were not externally validated. The results therefore do not establish transportability to other populations or healthcare systems, and the models should not yet be considered ready for routine clinical use. Finally, additional prognostic modalities—including quantitative electroencephalography, diffusion-weighted magnetic resonance imaging, and emerging biomarkers such as neurofilament light chain—were not systematically available within the registry and therefore could not be incorporated into the present prediction model. Because participating centers used different commercial assays for NSE measurement, inter-assay variability cannot be excluded. Furthermore, the registry did not contain all variables required to directly calculate and compare the BDCA and BDR scores. Individual-level data could not be made publicly available because of patient privacy and multicenter data-governance restrictions.

### 4.7. Future Directions

Future prospective studies should externally validate the proposed multimodal PTBD prediction model across diverse healthcare systems with different end-of-life practices. In addition, integration of automated quantitative CT analysis, artificial intelligence-assisted image interpretation, continuous electrophysiological monitoring, and novel blood biomarkers may further improve early identification of patients progressing toward brain death. The present study used standardized manual GWR measurements because validated automated measurements were not available in the registry during the study period. Automated approaches may improve reproducibility and clinical scalability but require prospective validation against standardized measurements and clinically relevant outcomes. Rather than replacing established multimodal neuroprognostication, these approaches may contribute to more individualized neurological risk stratification and precision neurocritical care after OHCA [17,18].

## 5. Conclusions

In conclusion, our study demonstrates that patients progressing toward brain death after out-of-hospital cardiac arrest represent a distinct neurological trajectory rather than simply the most severe subgroup of poor neurological outcomes. A multimodal prediction model integrating quantitative brain computed tomography, serum neuron-specific enolase measured at 48 h, and established clinical variables demonstrated good discrimination after internal validation for identifying patients at high risk of operationally defined PTBD. The incremental value of multimodal integration was particularly evident among patients with poor neurological outcomes, in whom the final model significantly outperformed both GWR and NSE alone. Rather than replacing guideline-recommended multimodal neuroprognostication, this model should be considered a complementary tool for early neurological risk stratification and individualized neurocritical care planning. Future prospective validation in diverse healthcare systems is warranted to confirm its generalizability and to determine whether earlier identification of patients following this clinical trajectory may facilitate a more individualized approach to neurocritical care after cardiac arrest.

## Figures and Tables

**Table 1:** Baseline and Clinical Characteristics According to Neurological Outcome and PTBD Status.

		Good Neurological Outcome	Poor Neurological Outcome			*P_all_*
			Non-PTBD	PTBD	*P_sub_*	
		(*n* = 88)	(*n* = 214)	(*n* = 74)		
Male		71 (80.7%)	144 (67.3%)	54 (73.0%)	0.445	0.061
Age		51.7 ± 14.5	64.0 ± 15.2	51.7 ± 16.8	<0.001	<0.001
Premorbid						
	Coronary artery disease	24 (27.3%)	38 (17.8%)	10 (13.5%)	0.507	0.063
	Hypertension	32 (36.4%)	91 (42.5%)	23 (31.1%)	0.110	0.19
	Diabetes mellitus	16 (18.2%)	62 (29.0%)	19 (25.7%)	0.694	0.15
	Cerebrovascular disease	3 (3.4%)	19 (8.9%)	5 (6.8%)	0.745	0.244
	Pulmonary disease	0 (0.0%)	29 (13.6%)	7 (9.5%)	0.475	0.001
Resuscitation variables						
	witnessed	68 (77.3%)	140 (65.4%)	41 (55.4%)	0.162	0.013
	bystander CPR	63 (71.6%)	133 (62.1%)	42 (56.8%)	0.496	0.129
	shockable rhythm	64 (72.7%)	46 (21.5%)	7 (9.5%)	0.033	<0.001
	Time to ROSC	20.6 ± 15.2	35.3 ± 21.5	42.3 ± 19.0	0.003	<0.001
	cardiac etiology	77 (87.5%)	123 (57.5%)	24 (32.4%)	<0.001	<0.001
	hanging injury	7 (8.0%)	19 (8.9%)	27 (36.5%)	<0.001	<0.001
Immediate after ROSC						
	Mean arterial pressure	98.2 ± 30.7	88.9 ± 31.5	87.2 ± 32.7	0.797	0.038
	GCS motor score >1	46 (52.3%)	29 (13.6%)	3 (4.1%)	0.043	<0.001
	convulsive movement	9 (10.2%)	45 (21.0%)	15 (20.3%)	1	0.079
	pupillary light reflex	61 (69.3%)	49 (22.9%)	10 (13.5%)	0.119	<0.001
	PH	7.2 ± 0.2	7.0 ± 0.2	7.0 ± 0.2	0.013	<0.001
	Lactic acid, mmol/L	7.8 ± 5.1	10.4 ± 5.8	11.9 ± 5.4	0.025	<0.001
	Glucose, mg/dl	245.0 ± 85.6	267.8 ± 129.5	294.5 ± 142.9	0.060	0.041
TTM related variables						
Pre-induction shock		24 (27.27%)	124 (57.94%)	51 (68.92%)	0.126	<0.001
Induction time, hrs		4.0 (2.0–5.7)	2.0 (1.0–3.9)	2.0 (1.0–3.8)	0.550	<0.001
Overshoot induction		21 (23.86%)	60 (28.04%)	13 (17.57%)	0.103	0.193
Post-hyperthermia		23 (26.14%)	27 (12.62%)	14 (18.92%)	0.252	0.016
Rearrest		9 (10.23%)	59 (27.57%)	12 (16.44%)	0.081	0.002
Tachycardia		18 (20.45%)	41 (19.25%)	14 (18.92%)	1.000	0.963
Bradycardia		5 (5.68%)	11 (5.14%)	0 (0.0%)	0.072	0.092
Hypoglycemia		5 (5.68%)	36 (16.82%)	7 (9.46%)	0.179	0.020
Hyperglycemia		36 (40.91%)	116 (54.21%)	46 (62.16%)	0.292	0.021
Hypokalemia		37 (42.05%)	88 (41.12%)	35 (47.30%)	0.430	0.647

Values are presented as *n* (%), mean ± standard deviation, or median (interquartile range), as appropriate. *P*_all_ indicates the overall comparison across the three outcome groups, and *P_sub_* indicates the comparison between the non-PTBD and PTBD groups among patients with poor neurological outcomes. Continuous variables were compared using one-way analysis of variance, Student’s *t*-test, the Kruskal–Wallis test, or the Mann–Whitney *U* test, as appropriate. Categorical variables were compared using the chi-square test or Fisher’s exact test. PTBD, progression toward brain death; CPR, cardiopulmonary resuscitation; ROSC, return of spontaneous circulation; GCS, Glasgow Coma Scale; TTM, targeted temperature management.

**Table 2:** Univariable and multivariable logistic regression analyses and performance of the final model for progression toward brain death in the total cohort (*n* = 376).

	Univariable		Multivariable	
	OR (95% CI)	*p*	aOR (95% CI)	*p*
Age (per year)	0.968 (0.953–0.984)	<0.001	0.948 (0.927–0.970)	<0.001
Resuscitation characteristics				
Witnessed	0.561 (0.334–0.944)	0.029	–	–
Bystander CPR	0.710 (0.423–1.190)	0.194	–	–
Shockable rhythm	0.182 (0.081–0.411)	<0.001	0.292 (0.109–0.780)	0.014
Time to ROSC (per minute)	1.023 (1.012–1.035)	<0.001	–	–
Cardiac etiology	0.245 (0.142–0.421)	<0.001	–	–
Hanging injury	6.098 (3.277–11.348)	<0.001	–	–
Clinical findings immediately after ROSC				
Initial pH	0.074 (0.022–0.248)	<0.001	–	–
Initial lactate (mmol/L)	1.069 (1.023–1.117)	0.003	–	–
Initial glucose (mg/dL)	1.002 (1.000–1.004)	0.044	–	–
Pre-induction shock	2.307 (1.343–3.965)	0.002	–	–
GCS motor score >1	0.128 (0.039–0.418)	0.001	–	–
Pupillary light reflex present	0.273 (0.135–0.553)	<0.001	–	–
Convulsive movement	1.168 (0.617–2.211)	0.634	–	–
Neuroprognostic markers				
NSE at 24 h	1.018 (1.012–1.024)	<0.001	–	–
NSE at 48 h	1.018 (1.013–1.023)	<0.001	1.016 (1.010–1.021)	<0.001
NSE at 72 h	1.016 (1.011–1.021)	<0.001	–	–
Low GWR (≤1.19)	14.457 (7.520–27.794)	<0.001	11.879 (5.617–25.121)	<0.001
Model performance			Final model	
Apparent AUC			0.895	
Optimism-corrected AUC			0.890	
Calibration slope			0.956	
Calibration intercept			−0.030	
Brier score			0.093	

Candidate predictors were first evaluated using univariable logistic regression. Adjusted odds ratios are presented only for variables retained in the final multivariable model. Dashes indicate variables not retained in the final model.

**Table 3:** Univariable and multivariable logistic regression analyses for progression toward brain death among patients with poor neurological outcomes (*n* = 288).

	Univariable		Multivariable	
	OR (95% CI)	*p*	aOR (95% CI)	*p*
Age (per year)	0.968 (0.953–0.984)	<0.001	0.949 (0.928–0.970)	<0.001
Resuscitation characteristics				
Shockable rhythm	0.382 (0.164–0.887)	0.025	–	–
Time to ROSC (per minute)	1.015 (1.003–1.027)	0.017	–	–
Cardiac etiology	0.355 (0.203–0.620)	<0.001	–	–
Hanging injury	5.896 (3.024–11.496)	<0.001	–	–
Clinical findings immediately after ROSC				
Initial pH	0.204 (0.057–0.724)	0.014	–	–
Initial lactate (mmol/L)	1.047 (1.000–1.096)	0.049	–	–
Initial glucose (mg/dL)	1.001 (1.000–1.003)	0.144	–	–
GCS motor score >1	0.270 (0.080–0.913)	0.035	–	–
Neuroprognostic markers				
NSE at 24 h	1.015 (1.009–1.020)	<0.001	–	–
NSE at 48 h	1.015 (1.010–1.020)	<0.001	1.014 (1.008–1.020)	<0.001
NSE at 72 h	1.013 (1.008–1.018)	<0.001	–	–
Low GWR (≤1.19)	9.063 (4.675–17.572)	<0.001	9.473 (4.484–20.012)	<0.001
Model performance			Final model	
Apparent AUC			0.864	
Optimism-corrected AUC			0.858	
Calibration slope			0.960	
Calibration intercept			−0.022	
Brier score			0.122	

Candidate predictors were first evaluated using univariable logistic regression. Adjusted odds ratios are presented only for variables retained in the final multivariable model. Dashes indicate variables not retained in the final model.

**Table 4:** Discriminative performance of quantitative GWR, serum NSE, and the multimodal model for prediction of progression toward brain death.

	AUC (95% CI)	Cut-Off	Sensitivity (95% CI)	Specificity (95% CI)	*p* vs. Final Model
**Total Cohort (*n* = 376)**					
GWR	0.858 (0.819–0.892)	≤1.19	85.1 (75.0–92.3)	75.5 (70.2–80.2)	0.054
		≤1.06	8.1 (3.0–16.8)	100.0 (98.8–100.0)	
NSE at 24 h	0.727 (0.679–0.771)	>45.2	58.1 (46.1–69.5)	81.8 (77.0–86.0)	<0.001
		>212.1	6.8 (2.2–15.1)	100.0 (98.8–100.0)	
NSE at 48 h	0.742 (0.695–0.786)	>77.7	52.7 (40.7–64.4)	90.1 (86.1–93.2)	<0.001
		>210.0	17.6 (9.7–28.2)	100.0 (98.8–100.0)	
NSE at 72 h	0.708 (0.659–0.754)	>59.4	43.2 (31.8–55.3)	88.1 (83.9–91.5)	<0.001
		>213.7	16.2 (8.7–26.6)	100.0 (98.8–100.0)	
Final model	0.895 (0.860–0.924)	>0.36	77.3 (65.8–86.0)	90.1(86.1–93.2)	Reference
**Poor Neurological Outcome (*n* = 288)**					
GWR	0.806 (0.756–0.850)	≤1.19	85.1 (75.0–92.3)	65.9 (59.1–72.2)	0.012
		≤1.06	8.1 (3.0–16.8)	100.0 (98.3–100.0)	
NSE at 24 h	0.684 (0.627–0.737)	>45.2	58.1 (46.1–69.5)	77.6 (71.4–83.0)	<0.001
		>212.1	6.8 (2.2–15.1)	100.0 (98.3–100.0)	
NSE at 48 h	0.691 (0.635–0.744)	>77.7	52.7 (40.7–64.4)	86.0 (80.6–90.3)	<0.001
		>210.0	17.6 (9.7–28.2)	100.0 (98.3–100.0)	
NSE at 72 h	0.661 (0.603–0.715)	>95.9	39.2 (28.0–51.2)	88.8 (83.8–92.7)	<0.001
		>213.7	16.2 (8.7–26.6)	100.0 (98.3–100.0)	
Final model	0.864 (0.819–0.901)	>0.31	74.3 (62.8–83.8)	88.3 (83.2–92.3)	Reference

Values for sensitivity and specificity are presented as percentages with 95% CIs. For each individual predictor, the first cut-off was selected using the Youden index, and the second cut-off was selected to provide 100% specificity. Pairwise comparisons of AUCs between the final multimodal model and each individual predictor were performed using DeLong’s test for correlated ROC curves. Reported *p* values represent comparisons with the final model. AUC, area under the receiver operating characteristic curve; CI, confidence interval; CT, computed tomography; GWR, gray-to-white matter ratio; NSE, neuron-specific enolase; ROC, receiver operating characteristic.

## Data Availability

Due to privacy restrictions, all data are stored at the researcher’s institution. Qualified researchers will be able to gain access via application to the corresponding author.
